# How Does Tube Size Affect Patients’ Experiences of Postoperative Sore Throat and Hoarseness? A Randomised Controlled Blinded Study

**DOI:** 10.3390/jcm10245846

**Published:** 2021-12-13

**Authors:** Pia Christiansen, Caroline Hornnes Pedersen, Hansjörg Selter, Lillian Odder, Jette Præstholm Riisager, Kjeld Damgaard, Signe Westmark, Niels Henrik Bruun, Dorte Melgaard

**Affiliations:** 1 Clinic of Anaesthesia, Aalborg University Hospital, 7700 Thisted, Denmark; pia.christiansen.1@rn.dk (P.C.); h.selter@rn.dk (H.S.); lillian.odder@gmail.com (L.O.); 2 Clinic of Anaesthesia, North Denmark Regional Hospital, 9800 Hjoerring, Denmark; c.hornnes@rn.dk (C.H.P.); jepr@rn.dk (J.P.R.); kad@rn.dk (K.D.); 3 Department of Clinical Medicine, Aalborg University, 9000 Aalborg, Denmark; 4 Centre for Clinical Research, North Denmark Regional Hospital, 9800 Hjoerring, Denmark; s.westmark@rn.dk; 5 Unit of Clinical Biostatistics and Epidemiology, Aalborg University Hospital, 9000 Aalborg, Denmark; nbru@rn.dk

**Keywords:** endotracheal intubation, upper airway anatomy, postoperative sore throat, postoperative pain, patient care

## Abstract

Sore throat (POST) and hoarseness (PH) are common complaints after endotracheal intubation (EI). The aim of this study was to investigate whether tube size impacts the experiences of POST and PH after EI in patients undergoing elective surgery, as well as to document a possible role of gender. This randomised, controlled, blinded study was conducted at Aalborg University Hospital, Thisted, Denmark or North Denmark Regional Hospital, Denmark. A total of 236 patients (53.4% female, mean age 50.9 years (SD 14.0)) were enrolled from the departments of gynaecology, parenchyma and orthopaedics. The patients were randomised to a tube size of 8.0 or 7.0 for males and 7.0 or 6.0 for females. Tube sizes were known to the anaesthesia staff but blinded for patients, researchers and staff at the postoperative care unit. POST and/or PH was reported 30–60 min before anaesthesia, at 30 min and at 2, 5, 12, 24, 48, 72 and 96 h after anaesthesia. Both female and male patients experienced significantly lower levels of POST and PH after intubation with the smallest tube size. This study demonstrates that a smaller size of tube results in a reduction in POST and PH after EI for both male and female patients.

## 1. Introduction

Minor complications and discomfort, such as postoperative sore throat (POST) and hoarseness (PH), are common following endotracheal intubation [1,2,3]. However, these complications may prolong recovery and cause patient dissatisfaction [4]. The incidence of POST is reported to be between 10 and 72%, while PH develops in 40–59% of patients [2,3,4,5,6,7].

Various studies have demonstrated that the most common factors associated with postoperative throat complaints are female gender, history of smoking, duration of anaesthesia, postoperative nausea, blood trail/stain on the endotracheal tube and natural teeth [6,8]. One study describes gender as a risk factor in the development of postoperative sore throat and hoarseness after endotracheal intubation [9]. However, the professional experience of the anaesthetic staff did not influence the incidence of POST [2,3]. Studies have documented that adjusting the endotracheal tube (ETT) cuff pressure to 20–30 cm H_2_O reduces POST and PH compared with the traditional pilot balloon palpation method [7,10]. A recent review documented that the tracheal tube size does not seem to affect the safety of anaesthesia or improve the risk of perioperative airway injury in adult patients undergoing elective surgery [11].

Evidence of tube sizes recommended for intubation is limited. However, the standard size seems to be 8.00 for males and 7.00 for females [12]. A randomised study documenting the effect of choosing a smaller tube size in elective surgical patients on POST and PH demonstrated a higher proportion of patients developing POST one–two hours postoperatively when intubated with a tube size of 7.0 versus a tube size of 6.0 [1]. Additionally, a systematic review and meta-analysis including 509 female patients documented that a tube size of 6.0 was associated with a lower incidence of POST [5]. One study described gender as a risk factor in the development of postoperative sore throat and hoarseness after endotracheal intubation [9].

The aim of this study was to investigate whether tube size impacts the experiences of POST and PH after endotracheal intubation in patients undergoing elective surgery, as well as to document a possible role of gender.

## 2. Materials and Methods

The study was performed in compliance with the Declaration of Helsinki. The North Denmark Region Committee on Health Research Ethics (N-20180078) and the Danish Data Protection Authority (2008-58-0028) approved the protocol. All study participants received oral and written information about the study and provided written informed consent. The study is registered on ClinicalTrials.gov (accessed on 4 October 2019) as NCT04184778.

From January 2019 to November 2020, 236 patients (53.4% female, mean age 50.9 years (SD 14.0)) undergoing elective surgery at Aalborg University Hospital, Thisted, Denmark or North Denmark Regional Hospital, Hjoerring, Denmark were included in this randomised, blinded, controlled study. The sample size was calculated on the basis of a previous study including 100 female patients, leading to the inclusion of at least 55 patients in each group [1]. Inclusion criteria were age ≥18 years, undergoing elective surgery with endotracheal intubation as the best airway management, American Society of Anaesthesiologists (ASA) 1–2, ability to read and understand Danish, access to a telephone for a postoperative follow-up interview. Patients were included from the departments of gynaecology, orthopaedics and gastrointestinal surgery. Exclusion criteria were current upper respiratory infection, confirmed dementia diagnosis, Simplified Airway Risk Index (SARI) > 4, Body Mass Index (BMI) > 35, rapid sequence induction and more than two attempts made for intubation.

**Figure 1:**
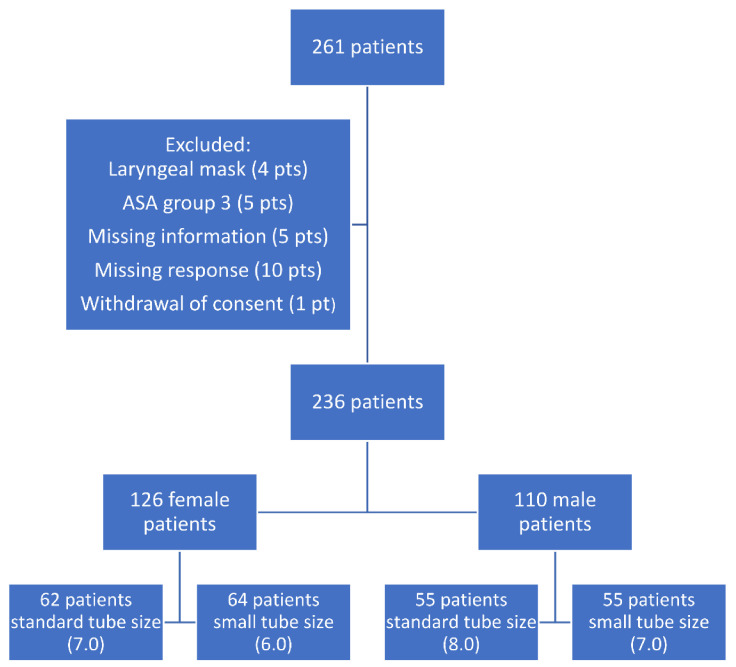
Flowchart of included patients.

The Research Electronic Data Capture (REDCap) application was used to randomise tube size, 6.0 or 7.0 for females and 7.0 or 8.0 for males, as illustrated in Figure 1. Tube sizes were known to the anaesthesia staff; patients, researchers and staff at the postoperative care unit (PACU) were blinded to tube size.

### 2.1. Procedure

Premedication followed the routines at the involved departments: paracetamol 1 g and a non-steroidal anti-inflammatory drug 400 mg. Patients with known postoperative nausea and vomiting (PONV) or at risk of PONV were treated with dexamethasone 4 mg and ondansetron 4 mg during anaesthesia. Local anaesthesia protocols at the two anaesthesia departments were followed, and based on either target controlled infusion, total intra venous anaesthesia with propofol 2–4 mg/kg and remifentanil 0.05 mg/mL, or inhalation with sevoflurane and fentanyl 0.1 mg/mL. Fentanyl 0.1 mg/mL was administered if necessary, at the end of surgery. All patients were intubated with EET with cuff (oral tracheal tube with Murphy eye from Unomedical distributed by Convatec, UK) size 6.0 or 7.0 for females and size 7.0 or 8.0 for males.

Patients were monitored with non-invasive blood pressure, 3-lead electrocardiography and pulse oximetry, and lungs were ventilated using a Primus Dräger Ventilator (Dräger Medical AG & Co, Lübeck, Germany). The ventilation modes were either pressure-controlled or volume-controlled depending on the patient’s physical status and the type of surgery. The standard procedure for treating postoperative pain at the PACU as well as at home was followed, and paracetamol, non-steroidal anti-inflammatory drugs and/or morphine were used. The tube was secured by air in the cuff and the cuff pressure was adjusted to be between 20 and 30 cm H_2_O and controlled twice during the anaesthesia with a cuff pressure gauge from Ambu A/S.

### 2.2. Data Collection

Data were collected by a registered nurse anaesthetist and entered into REDCap hosted at North Denmark Region [13,14]. Variables describing the intubation and extubation process, as well as the intraoperative factors and drugs relevant according to POST and PH, were collected (Table 1).

All participants reported their experience of POST and/or PH 30–60 min before anaesthesia, at 30 min and two hours after anaesthesia and again at five hours, 12, 24, 48, 72 and 96 h after anaesthesia. At discharge from the PACU, the patients received a questionnaire to report their experiences of POST and PH. They were contacted by an experienced nurse by telephone 8–12 days after discharge to report their answers. The patients were asked to report their subjective experience of POST and PH on a 4-step Likert scale: 0 = no sore throat, 1 = mildly sore throat (less pain than a common cold, 2 = moderately sore throat (like a common cold) and 3 = severely sore throat (more serious than a common cold). PH was rated using a similar scale.

### 2.3. Statistical Analysis

Analysis and data management were performed in Stata version 16. Baseline values as well as operation characteristics are summarised in tables. Continuous data are reported with mean and standard deviations and comparison by tube size was made by ANOVA. Categorical data are reported as a count and column percentages, and comparison by tube size was made using a chi-square test. Time-adjusted relative risks of sore throat and hoarseness were estimated from a Poisson regression with variance estimates using robust cluster estimation by person.

## 3. Results

The 236 included patients scheduled for elective surgery were recruited from the departments of gynaecology (*n* = 88), parenchyma (*n* = 85) and orthopaedics (*n* = 63). Patient characteristics assigned to the small versus standard tube size in the entire group of patients and in females and males, respectively, are summarised in Table 2. No significant differences were seen between groups.

**Figure 2:**
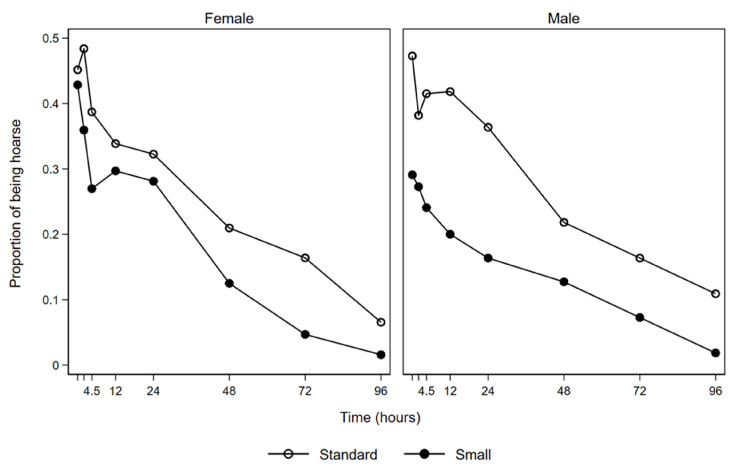
Proportion of patients experiencing hoarseness over time.

Figure 2 shows that both males and females experienced less hoarseness over time when the smaller tube sizes were used.

**Figure 3:**
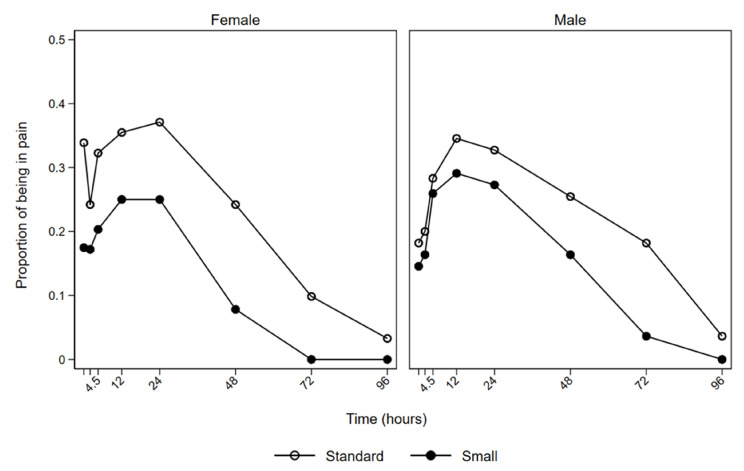
Proportion of patients experiencing pain over time.

Figure 3 illustrates that both males and females experienced less pain when the smaller tube sizes were used.

The time-adjusted relative risk estimates (Table 3) showed that females experienced a significant reduction in sore throat of 0.56 [0.35; 0.90] when a smaller tube size was applied and a non-significant reduction in hoarseness of 0.75 [0.51; 1.11]. Males experienced a significant reduction in hoarseness of 0.55 [0.34; 0.87] and a non-significant reduction in sore throat of 0.74 [0.43; 1.26].

## 4. Discussion

The aim of the present study was to investigate how tube size influences experiences of POST and PH after endotracheal intubation in patients undergoing elective surgery and to document a possible role of gender. The results showed that females intubated with a tube size of 6.0 versus a tube size of 7.0 and males intubated with a tube size of 7.0 versus 8.0 experienced significantly lower levels of POST and PH after intubation. Our results confirm the limited existing evidence of the effect of reducing the tube size on POST and PH [1,5]. Gender differences were documented according to the severity of POST and PH, but also according to the duration for which patients experienced POST and PH after intubation. These results confirmed a gender difference [9]. Moreover, it was also documented that some patients experienced POST and PH up to 96 h after surgery.

The current study has several strengths. One is the study design, with randomisation and blinding of the patients. Another strength is that the cuff pressure was maintained between 20 and 30 cm H_2_O for all patients. Other studies have documented that the cuff pressure can be a risk factor for the development of POST and PH; this was prevented in our study [7,10]. Guidelines were followed in all procedures. It has previously been documented that by measuring patients’ experiences of POST and PH two hours post-operatively, there is a risk of a residual effect of anaesthetics affecting the reported results [1]. In the present study, POST and PH were measured several times to avoid this risk, but a limitation is that follow-up stopped after 96 h, despite some patients still showing complaints of POST and PH at that time. However, the study has some weaknesses. More anaesthesiologists were involved in the inclusion of patients, and we had no record of whether all relevant patients complied with the inclusion and exclusion criteria. Similarly, we did not have information on patients who declined to participate. There were no major differences between the groups, thus eliminating the notion that the reduction in POST and PH might be related to the duration of anaesthesia, PONV or bloodstain/trail on the endotracheal tube, as reported factors related to POST and PH in other studies [6,8]. The statistical power of the present study is limited, and in order to confirm and expand the findings of our study, trials with a larger number of patients should be conducted.

In conclusion, this study suggests that a new practice is needed. Future directions of the present study should include females to be intubated with a tube size of 6.0 and males with a tube size of 7.0 to reduce POST and PH. It should be included in the patient information that patients may experience pain related to POST and PH up to 96 h after discharge.

The knowledge that a relatively large proportion of these patients who are discharged soon after surgery experience increased pain after discharge and up to 96 h after the operation should lead to an update of the patient information.

## 5. Conclusions

The main findings of this study were that a smaller size of EET results in more comfort for both male and female patients in terms of experiencing a sore throat or hoarseness after elective surgery. Further studies are needed to document the effects of using an even smaller tube size.

## Figures and Tables

**Table 1:** Collected variables.

Independent Variables		Intraoperative Variables	
Gender	Male/female	CUFF pressure 1	cm H_2_O
Age	Years	CUFF pressure 2	cm H_2_O
Length	Cm	Cough during intubation	Yes/no
Weight	Kg	Use of stilet	Yes/no
Smoker	Yes/no	Use of ventricular probe	Yes/no
ASA Score	Grade 1–2	Visible blood trail at the tube at extubation	Yes/no
SARI Score	Grade 0–4	Experience of the anaesthetic staff	Years
		Duration of the anaesthesia	Minutes
		PONV prophylaxes with dexamethasone 4 mg iv	Yes/no
		PONV prophylaxes with ondansetron 4 mg iv	Yes/no

**Table 2:** Patient and surgery characteristics.

	Total *N* = 236	Female *N* = 126			Male *N* = 110		
		Size 7.0 *n* = 62	Size 6.0 *n* = 64	*p*-Value	Size 8.0 *n* = 55	Size 7.0 *n* = 55	*p*-Value
Gender (Female)	126 (53.4)						
Age (years)	50.9 (14.0)	44.8 (12.4)	47.0 (12.4)	0.33	55.5 (12.7)	57.6 (14.4)	0.41
BMI (kg/m^2^)	27.1 (4.3)	27.6 (4.9)	26.8 (5.2)	0.37	27.4 (3.5)	26.6 (3.3)	0.21
Smoker (yes)	172 (73.2)	40 (65.6)	50 (78.1)	0.12	43 (78.2)	39 (70.9)	0.38
ASA score							
1	105 (44.5)	34 (54.8)	33 (51.6)		18 (32.7)	20 (36.4)	
2	131 (55.5)	28 (45.2)	31 (48.4)	0.71	37 (67.3)	35 (63.6)	0.69
SARI score							
0	156 (66.4)	40 (64.5)	47 (73.4)		34 (61.8)	35 (64.8)	
1	22 (9.4)	5 (8.1)	3 (4.7)		7 (12.7)	7 (13.0)	
2	35 (14.9)	10 (16.1)	9 (14.1)		7 (12.7)	9 (16.7)	
3	13 (5.5)	2 (3.2)	4 (6.3)		5 (9.1)	2 (3.7)	
4	9 (3.8)	5 (8.1)	1 (1.6)	0.35	2 (3.6)	1 (1.9)	0.76
CUFF pressure 1	25.1 (11.2)	27.0 (11.9)	25.6 (9.8)	0.48	23.6 (11.9)	23.7 (10.9)	0.96
CUFF pressure 2	22.8 (9.1)	23.9 (7.7)	23.6 (8.1)	0.81	21.8 (10.1)	21.8 (10.8)	0.99
Cough during intubation (yes)	34 (14.5)	9 (14.5)	8 (12.5)	0.74	9 (16.4)	8 (14.8)	0.82
Use of stilet (yes)	62 (26.3)	15 (24.2)	16 (25.0)	0.92	18 (32.7)	13 (23.6)	0.29
Use of ventricular probe (yes)	121 (52.2)	45 (72.6)	41 (65.1)	0.37	17 (32.1)	18 (33.3)	0.89
Visible blood trail at the tube at extubation (yes)	16 (7.0)	5 (8.1)	4 (6.6)	0.75	3 (5.7)	4 (7.4)	0.71
Experience of the anaesthetic staff (years)	13.7 (10.2)	12.2 (9.9)	13.1 (10.1)	0.60	16.7 (10.6)	13.3 (9.7)	0.08
Duration of the anaesthesia (min)	86.0 (43.5)	97.0 (49.3)	84.3 (50.8)	0.16	80.5 (32.5)	80.9 (34.7)	0.96
PONV prophylaxes with dexamethasone 4 mg iv (yes)	69 (29.6)	25 (41.7)	26 (41.3)	0.96	8 (14.5)	10 (18.2)	0.61
PONV prophylaxes with ondansetron 4 mg iv (yes)	77 (32.8)	29 (47.5)	29 (45.3)	0.80	9 (16.4)	10 (18.2)	0.80

**Table 3:** Time-adjusted relative risk estimates with 95% confidence intervals.

		RR	Lower 95% CI	Upper 95% CI	*p*-Value
	Females				
**Experiencing pain**	Tube size (standard)	1.00	1.00	1.00	
	Tube size (small)	0.56	0.35	0.90	0.02
**Experiencing hoarseness**	Tube size (standard)	1.00	1.00	1.00	
	Tube size (small)	0.75	0.51	1.11	0.16
	**Males**				
**Experiencing pain**	Tube size (standard)	1.00	1.00	1.00	
	Tube size (small)	0.74	0.43	1.26	0.27
**Experiencing hoarseness**	Tube size (standard)	1.00	1.00	1.00	
	Tube size (small)	0.55	0.34	0.87	0.01

## Data Availability

Data presented in this study are available on request from the corresponding author.
